# The left gastro-omental vessels are able to maintain the entire spleen blood supply

**DOI:** 10.1590/1677-5449.20210178

**Published:** 2022-04-22

**Authors:** Andy Petroianu

**Affiliations:** 1 Universidade Federal de Minas Gerais – UFMG, Faculdade de Medicina, Belo Horizonte, MG, Brasil.

**Keywords:** wandering spleen, splenomegaly, gastro-omental vessels, inferior polar vessels, blood supply, baço ectópico, esplenomegalia, vasos gastromentais, vasos polares inferiores, suprimento sanguíneo

## Abstract

The spleen is supplied by blood flow through the splenic artery and vein. The purpose of this communication is to report an ectopic spleen supplied only by reverse flow through the left gastro-omental vessels. A 14-year-old boy presented with pelvic splenomegaly supplied only by the left gastro-omental artery and veins connected to the inferior polar vessels, which were the only vessels communicating with the spleen. After detorsion of the spleen and splenopexy, the spleen returned to normal dimensions. The patient had uneventful follow-up. In conclusion, the left gastroepiploic vessels are able to maintain the entire spleen blood supply.

## Resumo

O baço é suprido pelo fluxo sanguíneo da artéria e veia esplênicas. O objetivo desta comunicação é apresentar um baço ectópico suprido apenas pelo fluxo sanguíneo reverso proveniente dos vasos gastromentais esquerdos. Um paciente de 14 anos apresentou esplenomegalia pélvica suprida apenas por artéria e veia gastromentais esquerdas, conectadas aos vasos polares inferiores, que eram os únicos presentes nesse baço. Após a distorção do baço e a esplenopexia, o baço voltou às dimensões normais. Não houve intercorrências no acompanhamento do paciente. Em conclusão, os vasos gastromentais esquerdos são capazes de suprir o fluxo sanguíneo de todo o baço.

## INTRODUCTION

The spleen is basically supplied by blood flow through the main splenic artery and vein. The splenic artery usually arises from the celiac trunk and divides into two or three branches before entering the hilum of the spleen. The superior and inferior branches of this artery are known as superior and inferior polar arteries. The superior pole of the spleen has an additional arterial supply, separate from the splenic hilar vessels, by mean of the splenogastric arteries, which connect the superior pole of the spleen to the gastric fundus.^1^^,^^2^ The inferior polar artery supplies the inferior splenic pole and is the origin of the left gastro-omental artery, which runs along the greater curvature of the stomach as an arch, anastomosing with the right gastro-omental artery, which is the continuity of the gastroduodenal artery.^1^^-^4

Blood from the parenchyma of the spleen flows through the superior and inferior polar veins, which join together to form the main splenic vein. Along its course, the inferior polar vein receives the left gastro-omental veins as tributaries, which drain blood from the greater omentum and the body of the stomach. The left gastro-omental veins run in the greater omentum as an arch and connect with the right gastro-omental veins, then continue as tributaries of the portal vein.^1^^,^^2^ The superior pole of the spleen has an additional venous drainage, separate from the splenic hilar vessels, by mean of the gastrosplenic veins, which connect the superior pole of the spleen to the gastric fundus.^1^^,^^2^

Splenoptosis (or free-floating, aberrant, ectopic, or wandering spleen) is a rare congenital condition defined as an ectopic spleen displaced from its normal anatomical position in the left hypochondrium due to absence of the perisplenic ligaments. This mobile spleen is supplied by a long vascular pedicle and may be associated with other congenital disorders.^5^^-^8

The purpose of this communication is to report the case of a spleen supplied only by reverse blood flow through the gastro-omental vessels.

The patient provided the authors with a signed written informed consent form for publication of this case report, including images. Ethical approval was not required; this paper reports treatment performed in a patient with no research intention.

## CASE REPORT

A 14-year-old boy presented with a pelvic mass associated with abdominal discomfort, dysuria, tenesmus, and constipation. A physical examination of the abdomen revealed a visible tender pelvic mass, changing position with variation of the patient’s decubitus. Axial computed tomography (CT) showed absence of the spleen in the left subphrenic space and a spleen-like mass in the pelvis, suggestive of a wandering spleen, compressing the bladder. Due to the abdominal discomfort and gradual increase in urinary and digestive symptoms, surgical treatment was recommended.

**Figure 1:**
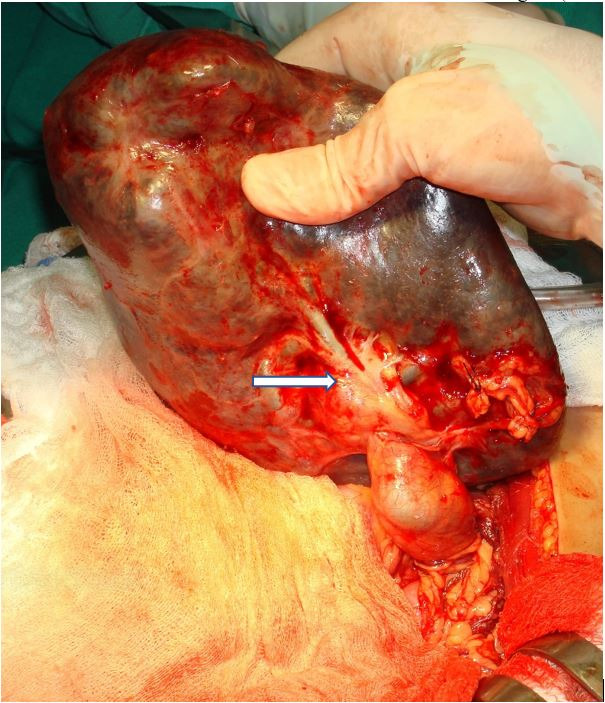
Surgical view of the wandering splenomegaly caused by splenic torsion. The spleen is only supplied by the left gastro-omental vessels of the greater omentum connected to the inferior polar artery and vein (arrow). Observe the absence of perisplenic ligaments and the lack of the main splenic artery and vein as well as of the superior polar and splenogastric vessels. Note the splenic congestion and the highly dilated inferior polar and left gastroepiploic veins due to the previous splenic torsion.

After opening the abdomen, an enlarged spleen measuring 22 × 16 × 13 cm with a long epiploic vascular pedicle and absence of perisplenic ligaments was found in the pelvis. The splenic pedicle comprised the greater omentum with the left gastro-omental artery and veins, starting at the tail of the pancreas. These vessels were partially twisted and connected to the inferior splenic polar artery and veins. Due to the splenic torsion, the organ was congested and both the gastro-omental and the inferior polar veins were extremely dilated. No other vessels were found in the splenic hilum (Figure 1).

The detectable arterial pulse was from the left gastro-omental artery to the splenic polar artery, characterizing reversed blood flow. The venous blood flow was also reversed from the inferior splenic polar vein to the left gastro-omental veins. The portal vein was formed by the superior and inferior mesenteric veins, associated with the left gastric vein, without the splenic vein. The body and tail of the pancreas were vascularized by vessels connected to the superior mesenteric artery and vein.

**Figure 2:**
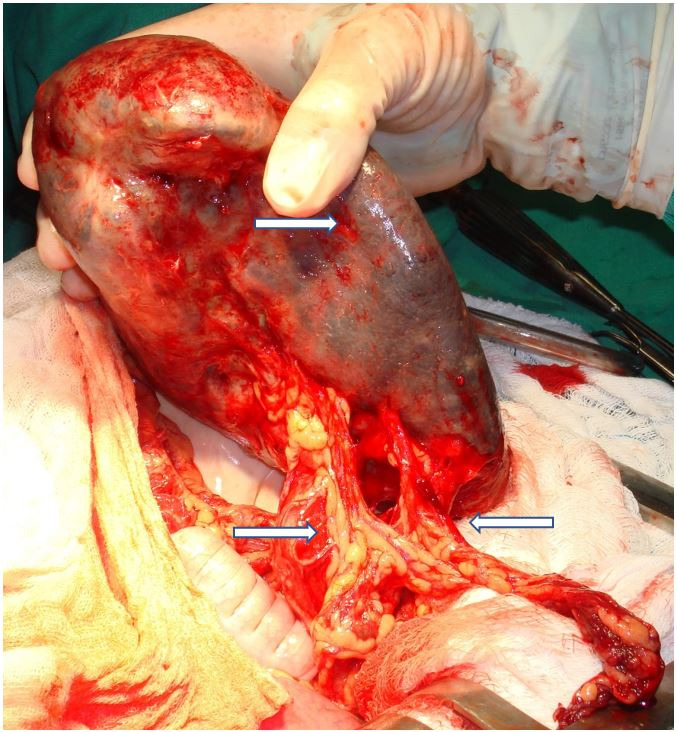
The same spleen shown in Figure 1 shortly after splenic detorsion. Observe that congestive splenomegaly has diminished and vein calibers have returned to normal dimensions (arrows).

After detorsion of the spleen, the venous calibers reduced to normal, the dimensions of the spleen reduced, and the signs of congestion disappeared (Figure 2). The spleen was relocated to the upper left quadrant and splenopexy was performed with four stitches to the diaphragm and left peritoneum, using 2-0 polyglactin 910 thread. The patient had an uneventful recovery and was discharged from the hospital on the second postoperative day. All urinary and digestive complaints as well as the abdominal discomfort disappeared after the surgical procedure. The four-year follow-up indicated no incidents related to the surgical procedure and the patient is still in good health living a normal life with no complaints related to the spleen, now in its normal location.

## DISCUSSION

An ectopic spleen is a rare condition, but more than 1000 papers have been published describing this disorder, which is most commonly found in women of reproductive age.^1^^,^^5^^-^8 Diagnosis is difficult because of nonspecific clinical or laboratory findings and is only confirmed by imaging exams.^7^^,^^8^ The condition is usually asymptomatic, but may be responsible for chronic abdominal pain associated with gastrointestinal and urinary symptoms. In fact, the purpose of this case report was not to present one more wandering spleen, but to emphasize the particular vascularization of this spleen, by the left gastro-omental vessels only. It is worthwhile to suppose that this study could contribute to challenging the concept that the role of the main splenic vessels is essential to spleen survival.

Splenogastric vessels are able to preserve the viability of the upper splenic pole if the splenic pedicle is damaged or must be ligated.^1^^,^^9^^-^15 Based on this knowledge, subtotal splenectomy, maintaining only the upper splenic pole supplied by the splenogastric vessels, has been performed to treat severe splenic trauma, portal hypertension, Gaucher’s disease, myeloid metaplasia, chronic lymphocytic leukemia, splenic hemangioma, splenic abscess, splenic pain due to thrombotic ischemia, somatic and sexual hypodevelopment due to splenomegaly, and pancreatic tail diseases.^1^^,^^10^^-^15 However, the splenogastric vessels are not able to supply the entire spleen.

Warshaw^16^ described distal pancreatectomy preserving the spleen supplied through the splenogastric and left gastro-omental vessels after resection of the splenic artery and vein together with the pancreatectomy. According to this author and colleagues, the splenogastric vessels are able to supply the upper part of the spleen. In contrast, the left gastro-omental vessels are able to maintain the viability of only the inferior pole of the spleen.^16^^-^18 Another procedure that interrupts the blood flow to the spleen is splenic artery embolization, used to treat an aneurysm of this artery or hypersplenism.^18^^-^20 In this case, the splenogastric and gastro-omental arteries supply the spleen.

Previously, we confirmed in a patient with Hodgkin’s lymphoma of the splenic superior margin that reverse blood flow of the gastro-omental vessels through the inferior splenic polar vessels is efficacious for preserving the inferior splenic pole after superior splenectomy.^21^ Nevertheless, no published study has described provision of splenic blood supply by the gastro-omental vessels alone.

Therefore, this is the first report that demonstrate the entire splenic blood supply by the left gastro-omental vessels only, through the inferior splenic pole artery and veins. These vessels were able to maintain the viability of an enlarged and congestive spleen even when partially twisted. No apparent infarction or other splenic vascular disorder was verified after detorsion of the spleen.

In the presence of a congenital disorder, other organic abnormalities must be investigated. In this case, the absence of the perisplenic ligaments was combined with absence of the splenic artery and vein, including the superior polar and splenogastric vessels.

## CONCLUSION

The left gastro-omental vessels are able to maintain the entire spleen blood supply.
